# Outcome and features of acute kidney injury complicating hypoxic hepatitis at the medical intensive care unit

**DOI:** 10.1186/s13613-016-0162-4

**Published:** 2016-07-08

**Authors:** Andreas Drolz, Thomas Horvatits, Kevin Roedl, Karoline Rutter, Katharina Staufer, Dominik G. Haider, Christian Zauner, Gottfried Heinz, Peter Schellongowski, Stefan Kluge, Michael Trauner, Valentin Fuhrmann

**Affiliations:** Division of Gastroenterology and Hepatology, Department of Internal Medicine III, Medical University of Vienna, Vienna, Austria; Department of Intensive Care Medicine, University Medical Center, Hamburg-Eppendorf, Martinistraße 52, 20246 Hamburg, Germany; Division of Transplantation, Department of Surgery, Medical University of Vienna, Vienna, Austria; Division of Nephrology, Department of Internal Medicine III, Medical University of Vienna, Vienna, Austria; Department of Emergency Medicine, Inselspital, University Hospital Bern, Bern, Switzerland; Intensive Care Unit 13H3, Division of Cardiology, Department of Internal Medicine II, Medical University of Vienna, Vienna, Austria; Intensive Care Unit 13I2, Division of Oncology and Infectious Diseases, Department of Internal Medicine I, Medical University of Vienna, Vienna, Austria

**Keywords:** Hypoxic hepatitis, Acute kidney injury, Renal replacement therapy, Mortality

## Abstract

**Background:**

Hypoxic hepatitis (HH) is a frequent and potentially life-threatening event typically occurring in critically ill patients as a consequence of hemodynamic impairment. While acute kidney injury (AKI) has been well described in patients with acute liver failure, incidence and outcome of AKI accompanying HH are unclear. The aim of this study was to assess incidence, clinical implications and outcome of AKI and renal replacement therapy (RRT) in critically ill patients with HH.

**Methods:**

A total of 1948 consecutive critically ill admissions were studied at the Medical University of Vienna. Laboratory and clinical parameters as well as the presence of HH and AKI were assessed on a daily basis. Outcome, renal recovery and length of stay were assessed and documented, and patients were followed for 1 year.

**Results:**

A total of 295 admissions (15 %) developed HH. Main precipitators were cardiogenic (44 %) and septic shock (36 %). Occurrence of HH was significantly associated with AKI [OR 4.50 (95 % CI 3.30–6.12)] and necessity of renal replacement therapy [RRT; OR 3.36 (95 % CI 2.58–4.37)], *p* < 0.001 for both. Two hundred forty admissions with HH (81 %) developed AKI, 159 of whom (66 %) had AKI stage 3. Both HH and AKI were significantly linked to mortality. AKI stage 3, international normalized ratio (INR, during HH) and the presence of septic shock were identified as independent predictors of 28-day mortality in admissions with HH, whereas RRT was identified as an independent protective factor. There was a synergistic effect of HH and AKI on length of stay at the ICU. Of all HH survivors treated with RRT, 71 % showed renal recovery during follow-up.

**Conclusion:**

HH is frequently complicated by occurrence of AKI. Severity of HH, AKI stage and the presence of septic shock seem to contribute to poor outcome in these patients. Initiation of RRT in HH with AKI may enable renal recovery and should not be withheld in medical ICU patients.

**Electronic supplementary material:**

The online version of this article (doi:10.1186/s13613-016-0162-4) contains supplementary material, which is available to authorized users.

## Background

Hypoxic hepatitis (HH) is a life-threatening complication accompanying states of oxygen depletion in critically ill patients at the intensive care unit (ICU) [1–5]. Incidence rates of approximately 10 % have been reported in critically ill patients at the medical ICU [3, 4, 6, 7], and mortality rate in these patients was more than 50 % [1, 4, 5]. Several risk factors for mortality in HH patients have been identified and include the presence of septic shock, sequential organ failure assessment (SOFA) score >10, international normalized ratio (INR) >2, jaundice and necessity of vasopressor therapy [1, 4, 5, 8].

Acute kidney injury (AKI) is a frequent complication in critical illness. As a consequence of various definitions being used over the last decades [9], incidence rates reported in the literature vary considerably ranging from 13 to 78 % in critically ill patients [10–15].

Yet, little is known about the frequency and features of acute kidney injury (AKI) in HH. Interestingly, some recommendations on the treatment of AKI in critically ill patients suggest not using renal replacement therapy (RRT) in patients with liver failure [9]. These recommendations are based on data obtained from cirrhotic patients with AKI and hepatorenal syndrome, respectively, and are therefore not necessarily applicable to patients with other kinds of liver failure such as HH. In contrast, Kidney Disease: Improving Global Outcomes (KDIGO) recommendations on AKI do not make a statement on patients with liver failure [16]. AKI in acute hepatic injury like HH may differ considerably from kidney injury accompanying chronic hepatic impairment or acute-on-chronic liver failure. This assumption is supported by the gradual recovery of renal function in all patients with AKI following acetaminophen-induced acute liver failure [17], thereby suggesting that renal failure in acute hepatic injury and acute liver failure is frequently reversible. However, to our knowledge, features and outcomes of AKI accompanying HH with respect to RRT have not been studied so far.

The aim of this study was to examine the prevalence, outcomes and renal recovery of AKI in patients with HH, the most frequent cause of severe acute hepatic impairment at the ICU. Furthermore, we aimed to assess the role of RRT in patients with HH and AKI.

## Methods

This study is based on a retrospective analysis of prospectively documented data including patient long-term follow-up between January 2005 and December 2011. All patients admitted consecutively to three medical ICUs at the Medical University Vienna were studied. This study was approved by the ethics committee of the Medical University Vienna, which waived the need for informed consent due to the observational character of this study.

On admission, simplified acute physiology score II (SAPS II) [18], SOFA [19], infections, organ dysfunctions (including need for vasopressor support, mechanical ventilation, RRT) and precipitating event for development of HH were documented. Routine laboratory analysis was conducted and documented on a daily basis.

All patients were screened on a daily basis for the presence of AKI defined by urine output and serum creatinine according to the criteria defined below [16], and patients were stratified according to the highest AKI stage observed during the ICU stay. RRT was started in patients with severe metabolic derangement, anuria irresponsive to fluids, hyperkalemia and/or uremic complications, as previously published [20]. In patients requiring RRT, SOFA score was calculated daily for the first 3 days following initiation of RRT. Type, duration and time of initiation of RRT were documented. Additionally, anticoagulation during RRT was documented. Recovery of renal function was assessed during follow-up.

HH was diagnosed according to the following well-established criteria [1, 3]: (a) a sharp but transient elevation of aminotransferase levels to at least 20-fold the upper limit of normal; (b) a setting of cardio-circulatory or respiratory failure; (c) exclusion of other potential causes of liver cell necrosis (viral or drug-induced hepatitis). If these criteria were met, a histologic confirmation was not required for the diagnosis. All patients were screened for the presence of HH by daily laboratory and clinical assessment starting from ICU admission until ICU discharge.

Cardiogenic shock was diagnosed in patients presenting with (a) systolic blood pressure <90 mmHg without the use of inotropes or vasopressors or requirement for vasopressors, (b) signs of decreased cardiac output (low cardiac output measured by any method, low mixed or central venous oxygen saturation), (c) clinical, echocardiographic or radiological signs of pulmonary congestion, (d) absence of hypovolemia and (e) signs of organ malperfusion (e.g., oliguria, cyanosis, lactate, change in mental status) [21, 22]. If available, noninvasive and invasive measurements of hemodynamics were considered for diagnosis, although these methods were not a prerequisite for diagnosis of cardiogenic shock [23, 24].

Septic shock was defined according to the recommendations of the surviving sepsis campaign [25].

Criteria for acute kidney injury were calculated according to the KDIGO clinical practice guideline for acute kidney injury based on serum creatinine and urinary output [16]. The value for baseline creatinine was obtained from the most recently measured creatinine concentration in the year prior to the ICU stay excluding the 7 days directly preceding ICU admission and considered to be representative of the baseline kidney function according to the judgment of the treating physician. If this baseline value was not available, the minimum serum creatinine at the time of admission to the ICU was used as baseline [10, 16, 26]. Renal recovery on hospital discharge was defined as independence from RRT [27].

ICU mortality, 28-day mortality and 1-year mortality were assessed on site or by contacting the patient or attending physician, respectively.

### Statistical analysis

SPSS Statistics 20 (IBM, Armonk, NY, USA) was used for statistical analysis. Continuous variables are expressed as median and interquartile range (IQR 25–75 %), binary variables as number (%). Univariate comparisons of continuous variables between groups were carried out using Mann–Whitney U test. Chi-square analysis and Fisher’s exact tests, as appropriate, were used to compare categorical variables. Risk factors for AKI in HH were assessed via ordinal logistic regression modeling.

A multivariate Cox regression model was developed in order to assess the impact of AKI and RRT, respectively, on 28-day survival in HH patients after adjustment for predefined confounders. Odds ratios (ORs) and hazard ratios (HRs) are presented with 95 % confidence intervals (95 % CI). A *p* value of less than 0.5 was considered statistically significant.

## Results

### Patients’ characteristics

A total of 1948 admissions were studied at three medical ICUs. Two hundred ninety-five admissions developed HH (15 %) during their stay at the ICU. In the vast majority (*n* = 229, 78 %), HH occurred within 24 h from ICU admission; 253 (86 %) developed HH within 48 h. Median time from ICU admission to occurrence of HH was 0 (IQR 0–1) days. Main precipitants for occurrence of HH were cardiogenic shock (*n* = 130; 44 %), septic shock (*n* = 106; 36 %), hemorrhagic shock (*n* = 8; 3 %) and mixed cardiogenic/septic shock (*n* = 3; 1 %). A detailed list of underlying conditions associated with occurrence of HH is shown in Additional file 1: Table S1. Two hundred forty-two admissions (12 %) had underlying liver cirrhosis. Clinical and laboratory features of 295 admissions with HH are illustrated in Table 1.

**Table 1:** Characteristics of 295 admissions with hypoxic hepatitis

Parameter	
Number of patients	295
Admission parameters	
Age (years), median (IQR)	63 (51–72)
Females,* n* (%)	94 (32 %)
SAPS II, median (IQR)	62 (45–79)
SOFA, median (IQR)	11 (8–15)
Heart rate (bpm), median (IQR)	81 (65–104)
MAP (mmHg), median (IQR)	68 (57–82)
Respiratory rate (breaths/min), median (IQR)	20 (16–25)
Body temperature (°C), median (IQR)	36.5 (35.5–37.5)
pH, median (IQR)	7.30 (7.20–7.40)
AST (IU/l), median (IQR)	366 (73–1265)
ALT (IU/l), median (IQR)	139 (39–706)
LDH (IU/l), median (IQR)	599 (360–1523)
INR, median (IQR)	1.4 (1.2–1.9)
Peak values	
AST (IU/l), median (IQR)	2457 (1168–5605)
ALT (IU/l), median (IQR)	1151 (534–2620)
LDH (IU/l), median (IQR)	2439 (1334–4999)
INR, median (IQR)	1.8 (1.4–2.7)
Underlying condition	
Cardiogenic shock, *n* (%)	130 (44 %)
Septic shock, *n* (%)	106 (36 %)
Mixed septic/cardiogenic shock, *n* (%)	3 (1 %)
Hemorrhagic shock, *n* (%)	8 (3 %)
Therapy	
Mechanical ventilation, *n* (%)	225 (76 %)
Vasopressor therapy, *n* (%)	231 (86 %)
Renal replacement therapy, *n* (%)	122 (41 %)
Outcome parameters	
28-day mortality, *n* (%)	170 (58 %)
ICU-LOS (days), median (IQR)	7 (2–14)

*SAPS II* simplified acute physiology score, *SOFA* sequential organ failure assessment, *MAP* mean arterial pressure, *AST* aspartate aminotransferase, *ALT* alanine aminotransferase, *LDH* lactate dehydrogenase, *ICU-LOS* length of stay at the ICU, *IQR* interquartile range

### Acute kidney injury and renal replacement therapy

Of all 1948 admissions studied during the observation period, 55 % (1054 admissions) developed AKI within 48 h after ICU admission. AKI stages 1, 2 and 3 occurred in 128 (7 %), 345 (18 %) and 581 (30 %) admissions to the ICU, respectively. Of those admissions with AKI stage 3, 70 % (*n* = 409) received RRT for a median of 4 (2–10) days. In total, 409 admissions (21 %) underwent RRT during their ICU stay with the median time from admission to initiation of RRT being 1 (IQR 0–3) day. A list of underlying reasons for initiation of RRT stratified according to the presence of HH is shown in Additional file 1: Table S2.

**Fig. 1:**
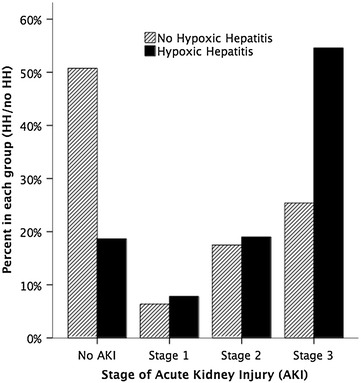
Distribution of AKI stages (KDIGO) in patients with and without hypoxic hepatitis. *HH* hypoxic hepatitis, *AKI* acute kidney injury—stratified according to the KDIGO criteria [16]

The distribution of AKI stages in admissions with and without HH is illustrated in Fig. 1. HH was significantly associated with AKI and RRT in the univariate analysis [OR 4.50 (95 % CI 3.30–6.12)] and [OR 3.36 (95 % CI 2.58–4.37); *p* < 0.0001 for both]. Two hundred forty admissions with HH (81 %) developed AKI, 159 of whom (66 %) had AKI stage 3. Risk factors for occurrence of AKI in HH are illustrated in Table 2.

**Table 2:** Univariate ordinal logistic regression model linking demographic, physiological and laboratory parameters to AKI in HH

Parameter	Overall	AKI Stage				*p* value*
	*n* = 295	0 (*n* = 55)	1 (*n* = 23)	2 (*n* = 56)	3 (*n* = 161)	
Baseline characteristics						
Age, years (IQR)	63 (51–72)	60 (45–71)	62 (50–77)	65 (54–71)	63 (52–71)	0.325
Sex (male), *n* (%)	201 (68 %)	31 (56 %)	19 (83 %)	35 (63 %)	116 (72 %)	0.086
SAPS II (IQR)	62 (45–79)	50 (33–68)	52 (37–73)	59 (38–75)	70 (52–86)	<0.001
SOFA (IQR)	11 (8–15)	10 (7–12)	9 (7–12)	10(6–13)	12 (8–16)	<0.001
Respiratory rate, bpm (IQR)	20 (16–25)	18 (15–22)	18 (15–27)	20 (16–25)	21 (17–27)	0.080
Body temperature, C° (IQR)	36.0 (34.8–36.8)	35.5 (33.6–36.5)	35.6 (33.5–36.5)	36.4 (35.2–36.9)	36.1 (35.0–36.8)	0.893
Heart rate, bpm (IQR)	81 (65–104)	79 (61–95)	83 (60–107)	79 (60–98)	83 (66–108)	0.119
Mean arterial pressure, mmHg (IQR)	68 (57–79)	73 (63–83)	72 (58–80)	70 (61–83)	65 (54–76)	<0.01
Preexisting liver cirrhosis,* n* (%)	22 (7 %)	1 (2 %)	0	5 (9 %)	16 (10 %)	<0.05
Cause of HH						
Cardiogenic shock,* n* (%)^a^	133 (45 %)	27 (49 %)	15 (65 %)	28 (50 %)	63 (39 %)	<0.05
Septic shock,* n *(%)^a^	109 (37 %)	12 (22 %)	5 (22 %)	14 (25 %)	78 (48 %)	<0.001
Hemorrhagic shock,* n* (%)	8 (3 %)	0	0	1 (2 %)	7 (4 %)	0.081
Therapy						
Mechanical ventilation,* n* (%)	225 (76 %)	36 (66 %)	19 (83 %)	35 (63 %)	135 (84 %)	<0.01
Vasopressor use, *n* (%)	247 (84 %)	38 (69 %)	18 (78 %)	35 (80 %)	146 (91 %)	<0.001
RRT, *n* (%)	122 (41 %)	0	0	0	122 (76 %)	<0.001
Laboratory parameters on admission						
Arterial lactate, mmol/l (IQR)	3.5 (1.7–8.2)	2.0 (1.2–4.4)	2.7 (1.6–5.7)	3.5 (2.1–6.9)	4.3 (2.1–10.6)	<0.001
Arterial pH (IQR)	7.30 (7.20–7.40)	7.39 (7.30–7.47)	7.32 (7.28–7.39)	7.32 (7.24–7.41)	7.27 (7.15–7.36)	<0.001
Sodium, mmol/l (IQR)	138 (133–142)	138 (133–140)	140 (136–143)	138 (133–141)	138 (133–142)	0.387
Potassium, mmol/l (IQR)	4.5 (3.9–5.2)	4.1 (3.6–4.7)	4.0 (3.7–4.4)	4.5 (4.1–5.1)	4.7 (4.1–5.5)	0.906
Albumin, g/l (IQR)	29.3 (24.1–34.0)	29 (24–32)	30 (26–36)	30 (24–35)	29 (24–34)	0.405
AST, U/l (IQR)	366 (73–1265)	345 (77–781)	474 (85–1341)	699 (108–1599)	248 (57–1475)	0.142
ALT, U/l (IQR)	139 (39–706)	129 (55–639)	219 (54–631)	217 (44–1470)	124 (34–682)	0.731
INR (IQR)	1.4 (1.2–1.9)	1.3 (1.1–1.6)	1.3 (1.1–1.4)	1.4 (1.2–1.9)	1.5 (1.2–2.1)	0.142
Bilirubin, mg/dl (IQR)	1.3 (0.7–2.7)	1.0 (0.6–2.1)	0.9 (0.7–1.6)	1.3 (0.8–3.1)	1.4 (0.7–3.2)	<0.05
Outcome						
28-day mortality, *n* (%)	170 (58 %)	17 (31 %)	6 (26 %)	25 (45 %)	122 (76 %)	<0.001

*SAPS II* simplified acute physiology score, *SOFA* sequential organ failure assessment, *HH* hypoxic hepatitis, *MCI* myocardial infarction, *RRT* renal replacement therapy, *AST* aspartate aminotransferase, *ALT* alanine aminotransferase, *INR* international normalized ratio, *IQR* interquartile range

* *p* values calculated using univariate ordinal logistic regression modeling with intercept

^a^Three patients had both cardiogenic and septic shock (mixed shock)

In admissions who developed HH, the percentage receiving RRT was significantly higher compared to critically ill admissions without HH (41 vs. 17 %, *p* < 0.001). This relationship is illustrated in a Cox-adjusted hazard plot (Additional file 2: Figure S1; adjusted for age, sex and SOFA score). Of all admissions with HH receiving renal support, 96 (79 %) had continuous RRT, 11 (9 %) underwent intermittent RRT, and 15 (12 %) had both continuous and intermittent types of RRT during their stay at the ICU.

Heparin (*n* = 80, 66 %) and citrate (*n* = 26, 21 %) were the most frequently used substances for anticoagulation. Epoprostenol and danaparoid were used in 5 (4 %) patients and 1 (1 %) patient, respectively. In 10 (8 %) admissions, the substance used for anticoagulation with RRT was changed during the stay at the ICU.

### Mortality in HH accompanied by AKI

**Fig. 2:**
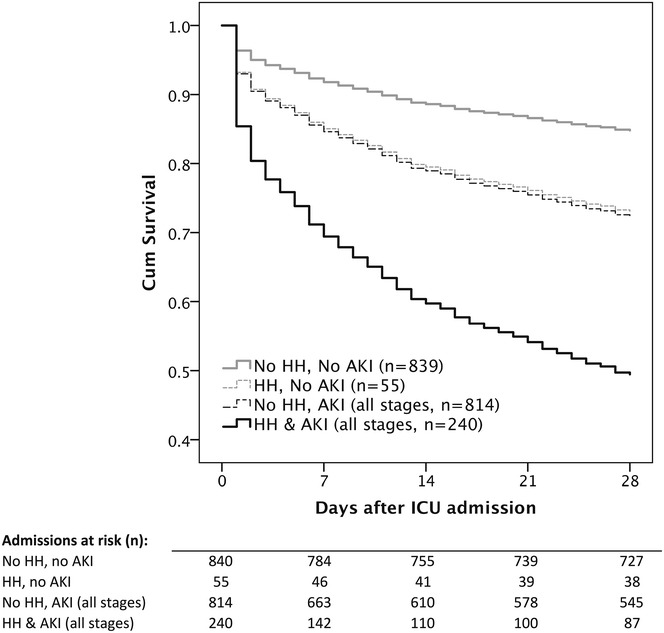
Cox-adjusted survival curves illustrating the impact of HH and AKI on survival in critically ill patients. *HH* hypoxic hepatitis, *AKI* acute kidney injury, *ICU* intensive care unit; adjusted for age, sex, sequential organ failure assessment (SOFA) score; significant differences between patients with HH and/or AKI compared to patients without HH or AKI (*p* < 0.05); adjusted hazard ratio (HR) for 28-day mortality in patients with HH and AKI was 4.26 (95 % CI 3.30–5.51), *p* < 0.001

Both HH and AKI were significantly linked to mortality as illustrated in Cox-adjusted survival curves (Fig. 2, adjusted for age, sex and SOFA score*)*. The worst outcomes, though, were observed in patients with the presence of HH and AKI (28-day mortality 64 %).

AKI stage 3, peak INR (within 48 h after occurrence of HH) and the presence of septic shock were identified as independent predictors of 28-day mortality in admissions with HH by multivariate Cox regression (Table 3). After adjustment for these factors, RRT was identified as an independent protective factor regarding 28-day mortality (Table 3).

**Table 3:** Cox regression model for 28-day mortality in HH

Parameter	Hazard ratio (95% CI)		
	Unadjusted	Adjusted^a^	Adjusted^b^
Age	1.01 (0.99–1.02)	1.01 (0.99–1.02)	1.00 (0.99–1.02)
Gender (male)	1.22 (0.87–1.70)	1.18 (0.83–1.67)	1.26 (0.86–1.84)
Admission SOFA score	1.08 (1.04–1.12)**	1.04 (0.99–1.07)	1.03 (0.99–1.07)
Septic shock	2.07 (1.53–2.80)**	2.15 (1.32–3.50)**	2.37 (1.41–3.97)**
Cardiogenic shock	0.86 (0.64–1.17)	1.62 (0.99–2.63)	1.94 (1.16–3.24)*
INR	1.29 (1.20–1.39)**	1.24 (1.14–1.34)**	1.26 (1.13–1.39)**
Acute kidney injury (AKI)			
AKI 1 vs. no AKI	0.83 (0.33–2.11)	0.77 (0.30–1.95)	0.78 (0.30–2.00)
AKI 2 vs. no AKI	1.69 (0.91–3.12)	1.50 (0.81–2.79)	1.55 (0.81–2.97)
AKI 3 vs. no AKI	3.48 (2.09–5.78)**	3.40 (1.85–6.24)**	3.51 (1.86–6.62)**
Renal replacement therapy	1.86 (1.37–2.51)**	0.64 (0.42–0.97)*	0.62 (0.39–0.97)*

*SOFA* sequential organ failure assessment, *INR* international normalized ratio, *AKI* acute kidney injury

* p<0.05, ** p<0.01

^a^Patients with chronic liver disease included—adjusted for all variables (total *n= 295* )

^b^Patients with chronic liver disease excluded—adjusted for all variables (total *n = 273*)

In HH patients requiring RRT, SOFA scores and number of organs failing during the first 3 days of RRT were associated with 28-day mortality (Additional file 1: Table S3). Persisting failure of ≥5 organs and/or SOFA scores above 20 for more than 48 h despite initiation of RRT was associated with a 100 % mortality rate.

Time from onset of AKI to initiation of RRT did not differ significantly between 28-day survivors and non-survivors [2 (0–7) days vs. 1 (0–3) days, *p* = 0.164]. No difference in 28-day mortality was observed between the two most frequently used methods of anticoagulation [heparin (*n* = 64, 80 %) vs. citrate (*n* = 19, 73 %); *p* = 0.457]. Similarly, incidence of bleeding complications did not differ between HH patients with heparin and citrate anticoagulation [*n* = 11 (14 %) vs. *n* = 3 (12 %), *p* = 0.772].

In the vast majority (*n* = 218, 91 %) of HH patients with AKI, AKI occurred within 48 h prior to or after HH. RRT was initiated after occurrence of HH in 54 patients (44 %), on the same day of HH in 32 patients (26 %), prior to HH in 36 patients (30 %).

### Length of stay at the ICU in HH and AKI

Overall length of stay (LOS) at the ICU did not differ significantly between HH admissions with and without AKI [6 (IQR 2–13) days vs. 8 (IQR 3–14) days; *p* = *0.295*], which was attributable to the high mortality rate in AKI accompanying HH. However, in ICU survivors, HH and AKI significantly affected ICU-LOS (Additional file 3: Figure S2). Median LOS in ICU survivors was 3 (2–8) days in admissions without HH or AKI, 9 (4–14) days HH without AKI, 5 (2–12) days in AKI without HH and 8 (5–20) days in admissions with both HH and AKI. Additionally, compared to admissions without HH and RRT, respectively, ICU-LOS more than doubled when HH was accompanied by need for RRT [4 (IQR 2–10) days vs. 10 (IQR 3–20) days], *p* < 0.0001.

### Renal recovery and long-term outcomes

Twenty-four admissions with HH and RRT survived their stay at the ICU. The ICU survival rate was significantly lower compared to admissions with RRT but without HH (20 % in patients with HH compared to 64 % in non-HH admissions, *p* < 0.001). Seventeen out of the 24 admissions (71 %) showed recovery of renal function at the time of hospital discharge. There was a trend for lower 1-year mortality in HH admissions with RRT and recovery of renal function compared to those without renal recovery [4 (24 %) vs. 4 (57 %), *p* = 0.167].

In admissions requiring RRT who survived the ICU stay (*n* = 207, 51 %), 1-year mortality rate did not differ significantly between those who had and who had not experienced HH [8 (33 %) vs. 68 (37 %), *p* = 0.715].

## Discussion

This is the first study that assesses features and outcomes of AKI and RRT in patients with HH compared to a large control group of critically ill patients. Our results demonstrate that HH is frequently accompanied by AKI. Most of the HH patients developed AKI stage 3. Importantly, occurrence of AKI was associated with increased mortality and length of stay in patients with HH.

Our study demonstrates that AKI is a frequent complication in critically ill patients associated with poor outcome [10–14]. In our total cohort, incidence rate of AKI was 55 % (and 49 % even in patients without HH), thus considerably higher than recently reported in the FINNAKI study (39 % incidence of AKI) [10]. However, incidence of AKI in our total cohort is comparable to recently published incidence rates according to KDIGO criteria in critically ill patients [26].

AKI was observed in the vast majority of HH patients with only 19 % preserving normal renal function. There was a temporal relationship between HH and AKI, and in most affected patients both events occurred early during the ICU stay. However, the temporal relationship varied between patients. AKI in HH was associated with severity of illness reflected by SAPS and SOFA score, but most importantly with shock, vasopressor use and admission lactate levels. Thus, ischemia and tissue hypoxia seem to play a crucial role in development of AKI in HH.

In accordance with other studies [10, 17], ICU stay was significantly prolonged in patients with AKI; 28-day mortality rate in patients with AKI was 40 % in the total study cohort. In contrast, AKI in HH was associated with significantly higher 28-day mortality rates exceeding 60 % by far. The high mortality rates are in part attributable to the severity of illness reflected by high SAPS and SOFA scores on ICU admission. Yet, AKI, the presence of septic shock and peak INR values during HH were independent predictors of mortality in HH patients. Furthermore, we observed a synergistic effect of HH and AKI resulting in longer stay and increased mortality at the ICU. One reason for that observation may be the described systemic effects of AKI, which may further impair liver function [28].

Initiation of RRT was identified as an independent protective factor regarding survival in patients with HH. While necessity of RRT was significantly associated with increased 28-day mortality in the univariate analysis, this effect was completely abolished after adjustment for demographics, AKI, septic shock, INR and severity of disease. Initiation of RRT may provide beneficial effects in patients with HH. Apart from regulation of fluid balance, metabolic stabilization and removal of water-soluble toxins may contribute to this hypothesis. Best timing for initiation of RRT in critically ill patients is still a matter of debate. A recent large randomized controlled study comparing early versus late initiation of RRT in critically ill patients could not observe a difference in outcome [29]. Yet, future studies are required to assess timing, modality and potential benefits of RRT in patients with HH complicated by AKI.

Renal recovery was observed in the majority (71 %) of ICU survivors with HH and necessity of RRT. This finding is comparable to patients with ALF, where similarly high rates of renal recovery in patients requiring RRT have been described [30].

On the other hand, we observed a 100 % mortality rate in admissions with persisting failure of ≥5 organs (or SOFA scores >20) despite use of RRT for at least 2 days. Thus, our data suggest that continuation of RRT and other therapeutic measures should be repeatedly reevaluated on an individual basis in critically ill HH patients who present with persisting 5- or 6-organ failure despite use of RRT for more than 48 h. Furthermore, future studies are needed to address the question of when to start and to stop RRT in patients with (persisting) multi-organ failure.

Consensus recommendations on prevention and management of AKI in critically ill patients suggest that RRT should not be used in patients with liver failure, who are not candidates for liver transplantation [9]. Yet, the characteristic laboratory pattern of HH presenting with high aminotransferase levels and increased INR frequently fulfills criteria of acute liver failure [2, 7, 31]. Thus, distinguishing HH from other types of acute liver injury is of crucial importance due to its different clinical pattern and therapeutic consequences. It has been suggested that “the ability to manage heart failure or other causes of ischemia successfully will determine outcome for these (HH) patients, and transplantation is seldom indicated” [31]. Given the high incidence rates of HH in critically ill patients [2, 4, 6], HH should be considered as one of the primary differential diagnoses in patients with massive elevation of aminotransferase levels in the setting of critical illness. Our data suggest that, although mortality is still very high, RRT should be initiated in patients with HH developing AKI. Thus, RRT should not be withheld in patients with unclear elevation of aminotransferase levels at the ICU until the etiology has been clarified.

This study has strengths and limitations. This study comprises one of the largest datasets on HH published so far and uncovered for the first time the previously unrecognized association with AKI. Furthermore, the control group (without HH) contains 1653 critically ill medical patients. This sample size enables a good characterization of AKI in HH as compared to AKI in patients without HH. Yet, this study was conducted at three medical ICUs. Thus, our data may not be transferable to surgical ICUs or wards. Residual confounding arising from unmeasured covariates cannot be entirely excluded. Thus, further studies in different samples are required.

## Conclusions

In sum, we could demonstrate that AKI frequently occurs in HH and is associated with high mortality and increased length of stay in survivors. Severity of the underlying disease and states of shock seem to contribute to AKI and poor outcome in HH. Initiation of RRT in HH with AKI may enable renal recovery and should not be withheld in medical ICU patients.

## Additional files

10.1186/s13613-016-0162-4 Supplementary Tables. **Table S1.** Major underlying conditions associated with occurrence of hypoxic hepatitis. **Table S2.** Underlying conditions in patients with and without hypoxic hepatitis at the time RRT was initiated. **Table S3.** SOFA score, organ failures and 28-day-mortality in HH patients requiring renal replacement therapy.
10.1186/s13613-016-0162-4 Cox-adjusted hazard plot illustrating the relation between occurrence of HH and requirement for RRT. HH means hypoxic hepatitis, RRT renal replacement therapy; Adjusted hazard ratio (HR) for RRT in HH was 2.25 (95 % CI 1.81–2.79), p < 0.001; adjusted for age, sex and SOFA score.
10.1186/s13613-016-0162-4 Percentage survivors remaining at the ICU. HH means hypoxic hepatitis, AKI acute kidney injury, ICU intensive care unit; pairwise log rank tests: p < 0.01 for all pairs, except HH & no AKI vs. HH & AKI (p = 0.207), and HH & no AKI vs. No HH & AKI (p = 0.304).

## Abbreviations

AKI: acute kidney injury
CI: confidence interval
HH: hypoxic hepatitis
HR: hazard ratio
ICU: intensive care unit
INR: international normalized ratio
IQR: interquartile range
KDIGO: Kidney Disease: Improving Global Outcomes
LOS: length of stay
OR: odds ratio
RRT: renal replacement therapy
SAPS: implified acute physiology score
SOFA: sequential organ failure assessment
