# Supplementary material for: Outcome and features of acute kidney injury complicating hypoxic hepatitis at the medical intensive care unit
Source: Ann Intensive Care. 2016 Jul 8;6:61. doi: 10.1186/s13613-016-0162-4 (PMC4938842; doi:10.1186/s13613-016-0162-4)
Supplement: Supplementary file 1 — 10.1186/s13613-016-0162-4 Supplementary Tables. Table S1. Major underlying conditions associated with occurrence of hypoxic hepatitis. Table S2. Underlying conditions in patients with and without hypoxic hepatitis at the time RRT was initiated. Table S3. SOFA score, organ failures and 28-day-mortality in HH patients requiring renal replacement therapy. [file 13613_2016_162_MOESM1_ESM.docx]

**SUPPLEMENTAL TABLES**

Table S1 Major underlying conditions associated with
occurrence of Hypoxic Hepatitis

| **Major underlying conditions associated wih occurrence of HH** | | |
| --- | --- | --- |
|  |  |  |
| **Cardiogenic/Cardiovascular** | |  |
|  | Myocardial infarction, n (%) | 68 (23%) |
|  | Pericardial effusion, n (%) | 13 (6%) |
|  | Valvular heart disease, n (%) | 48 (16%) |
|  | Rhythmogenic heart disease, n (%) | 58 (20%) |
|  | Cardiopulmonary resuscitation, n (%) | 77 (26%) |
|  | Cardiomyopathy, n (%) | 71 (24%) |
|  | Right heart failure, n (%) | 30 (10%) |
|  | Patients with multiple (> 1) conditions ^b^ | 117 (40%) |
|  | Cardiogenic shock, n (%)^a^ | 133 (45%) |
|  |  |  |
| **Infection/Sepsis** | |  |
|  | Pneumonia, n (%) | 57 (19%) |
|  | Urinary tract infection, n (%) | 3 (1%) |
|  | Soft tissue infections, n (%) | 5 (2%) |
|  | Endo-/myocarditis, n (%) | 10 (3%) |
|  | Meningitis/encephalitis, n (%) | 4 (1%) |
|  | Gastrointestinal, n (%) | 23 (8%) |
|  | Blood stream, n (%) | 12 (4%) |
|  | Other, n (%) | 6 (2%) |
|  | Infection of unknown origin, n (%) | 12 (4%) |
|  | Patients multiple (> 1) conditions ^b^ | 5 (2%) |
|  | Septic shock, n (%)^a^ | 109 (37%) |
| ^a^ 3 patients suffered from both cardiogenic and septic shock; | | |

Table S2 Underlying conditions in patients with and without Hypoxic Hepatitis at the time RRT was initiated

| **Underlying conditions at time of RRT-start** | **HH (n=122)** | **No HH (n=287)** |
| --- | --- | --- |
| Metabolic derangement/acidosis, n (%) | 92 (75%) | 116 (40%) |
| Hyperkalemia, n (%) | 52 (42%) | 69 (24%) |
| Anuria, n (%) | 43 (35%) | 96 (33%) |
| Uremia/Azotemia, n (%) | 115 (94%) | 247 (86%) |
|  |  |  |
| ≥ 2 conditions, n (%) | 115 (94%) | 176 (61%) |
| ≥ 3 conditions, n (%) | 54 (44%) | 56 (20%) |
| Acidosis: pH < 7.35, Hyperkalemia: potassium > 5 mmol/l, Anuria: urin productuion < 100ml/24h; Azotemia: creatinine and/or blood urea nitrogen higher than 1.5-times the upper limit of normal, Uremia: azotemia with clinical symptoms suggestive of uremia; RRT means renal replacement therapy | | |

Table S3 SOFA score, organ failures and 28-day-mortality in
HH patients requiring renal replacement therapy

| **Parameter** | | | **Overall** | **28-day-survivors** (n=31) | **28-day non-survivors**  (n=91) | **p-value** |
| --- | --- | --- | --- | --- | --- | --- |
|  |  |  |  |  |  |  |
| **Initiation of RRT (Day 1)** (n=122) | | |  |  |  |  |
|  | SOFA, *median (IQR)* | | 17 (15-18) | 15 (13-17) | 17 (15-19) | <0.01 |
|  | Nr. of organs failing, *median (IQR)* | | 4 (3-4) | 3 (3-4) | 4 (3-4) | <0.05 |
|  |  |  |  |  |  |  |
| **24 hours after initiation of RRT** (n=112) | | |  |  |  |  |
|  | SOFA, *median (IQR)* | | 17 (15-19) | 15 (13-17) | 18 (16-20) | <0.001 |
|  | Nr. of organs failing, *median (IQR)* | | 4 (3-4) | 3 (3-4) | 4 (4-5) | <0.01 |
|  |  |  |  |  |  |  |
| **48 hours after initiation of RRT** (n=93) | | |  |  |  |  |
|  | SOFA, *median (IQR)* | | 17 (15-19) | 15 (13-17) | 18 (16-20) | <0.001 |
|  | Nr. of organs failing, *median (IQR)* | | 4 (3-5) | 3 (2-4) | 4 (3-5) | <0.001 |
| RRT means renal replacement therapy, SOFA sequential organ failure assessment, IQR interquartile range | | | | | | |
