# Supplementary figures and images for: Outcome and features of acute kidney injury complicating hypoxic hepatitis at the medical intensive care unit
Source: Ann Intensive Care. 2016 Jul 8;6:61. doi: 10.1186/s13613-016-0162-4 (PMC4938842; doi:10.1186/s13613-016-0162-4)

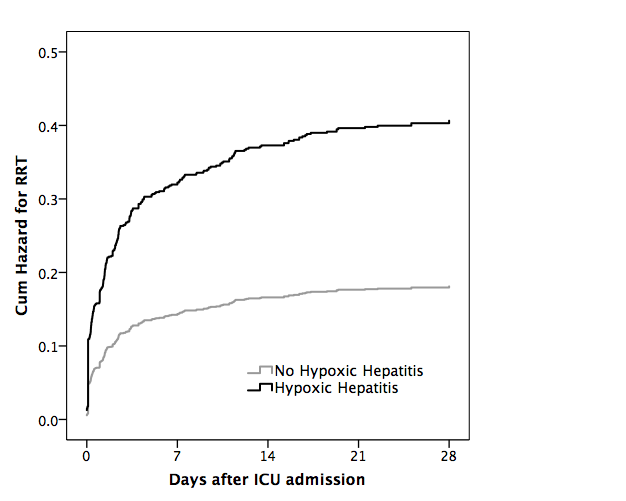

Supplement: Supplementary file 2 — 10.1186/s13613-016-0162-4 Cox-adjusted hazard plot illustrating the relation between occurrence of HH and requirement for RRT. HH means hypoxic hepatitis, RRT renal replacement therapy; Adjusted hazard ratio (HR) for RRT in HH was 2.25 (95 % CI 1.81–2.79), p < 0.001; adjusted for age, sex and SOFA score. [file 13613_2016_162_MOESM2_ESM.tiff]

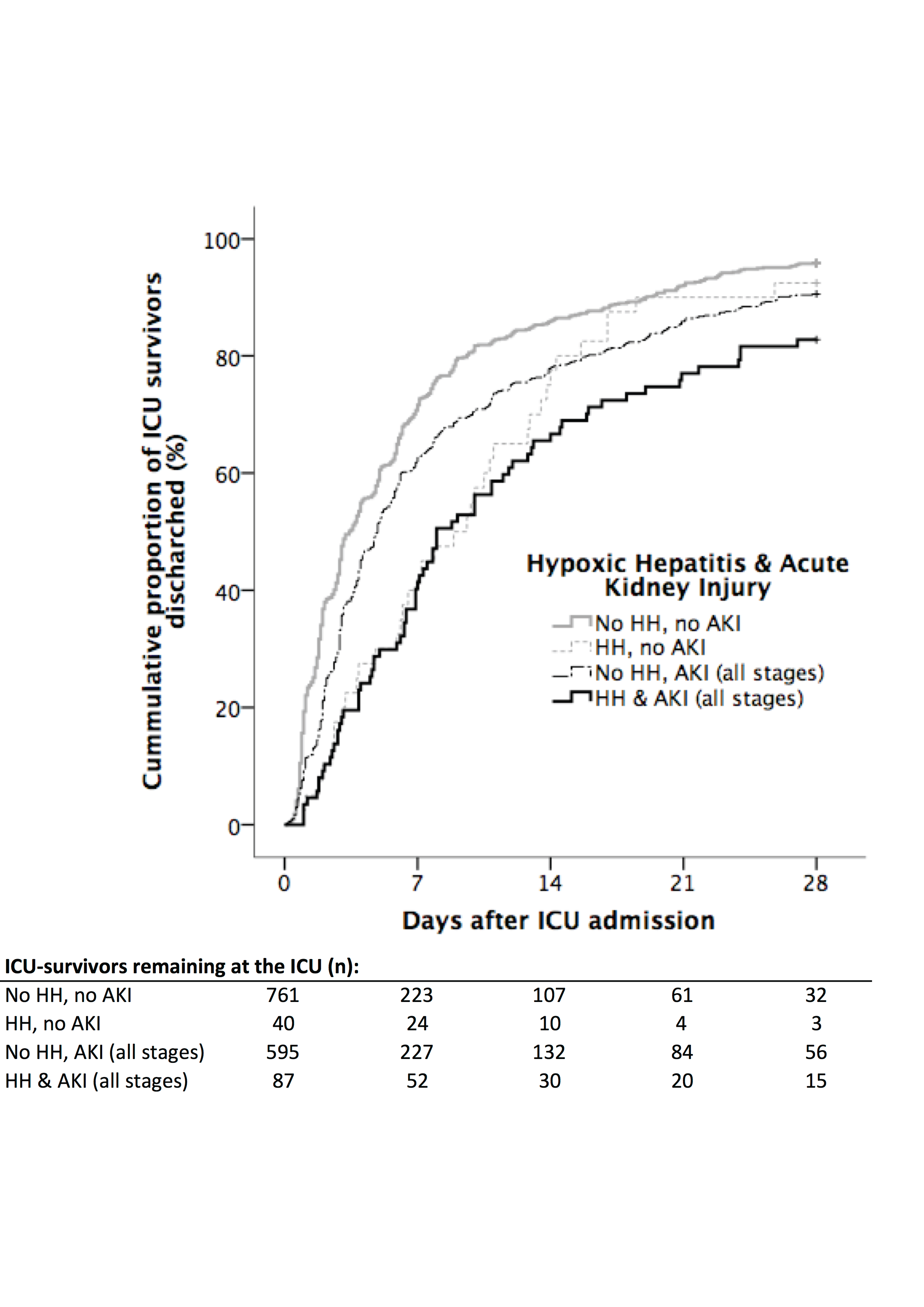

Supplement: Supplementary file 3 — 10.1186/s13613-016-0162-4 Percentage survivors remaining at the ICU. HH means hypoxic hepatitis, AKI acute kidney injury, ICU intensive care unit; pairwise log rank tests: p < 0.01 for all pairs, except HH & no AKI vs. HH & AKI (p = 0.207), and HH & no AKI vs. No HH & AKI (p = 0.304). [file 13613_2016_162_MOESM3_ESM.tiff]
